# Comparative analysis of the gut microbiota composition between knee osteoarthritis and Kashin-Beck disease in Northwest China

**DOI:** 10.1186/s13075-022-02819-5

**Published:** 2022-05-30

**Authors:** Yujie Ning, Minhan Hu, Yi Gong, Ruitian Huang, Ke Xu, Sijie Chen, Feiyu Zhang, Yanli Liu, Feihong Chen, Yanhai Chang, Guanghui Zhao, Cheng Li, Rong Zhou, Mikko J. Lammi, Xiong Guo, Xi Wang

**Affiliations:** 1 School of Public Health, Xi’an Jiaotong University Health Science Center, Key Laboratory of Trace Elements and Endemic Diseases, National Health and Family Planning Commission, Xi’an, Shaanxi 710061 People’s Republic of China; 2 Department of Joint Surgery, Hong Hui Hospital, Xi’an Jiaotong University, No.555, Youyi East Road, Xi’an, People’s Republic of China; 3 Shaanxi Provincial People’s Hospital, Xi’an, People’s Republic of China; 4 Shaanxi Institute of Endemic Disease Prevention and Control, Xi’an, Shaanxi 710003 People’s Republic of China; 5 Department of Integrative Medical Biology, University of Umeå, Umeå, Sweden; 6 Department of Occupational and Environmental Health, School of Public Health, Xi’an Jiaotong University Health Science Center, Xi’an, Shaanxi 710061 People’s Republic of China; 7 Global Health Institute, Xi’an Jiaotong University Health Science Center, Xi’an, Shaanxi 710061 People’s Republic of China

**Keywords:** Osteoarthritis, Kashin-Beck disease, 16S rDNA sequencing, Metagenomic sequencing

## Abstract

**Background:**

Osteoarthritis (OA) and Kashin-Beck disease (KBD) both are two severe osteochondral disorders. In this study, we aimed to compare the gut microbiota structure between OA and KBD patients.

**Methods:**

Fecal samples collected from OA and KBD patients were used to characterize the gut microbiota using 16S rDNA gene sequencing. To identify whether gut microbial changes at the species level are associated with the genes or functions of the gut bacteria between OA and KBD groups, metagenomic sequencing of fecal samples from OA and KBD subjects was performed.

**Results:**

The OA group was characterized by elevated *Epsilonbacteraeota* and *Firmicutes* levels. A total of 52 genera were identified to be significantly differentially abundant between the two groups. The genera *Raoultella*, *Citrobacter*, *Flavonifractor*, *g__Lachnospiraceae_UCG-004*, and *Burkholderia-Caballeronia-Paraburkholderia* were more abundant in the OA group. The KBD group was characterized by higher *Prevotella_9*, *Lactobacillus*, *Coprococcus_2*, *Senegalimassilia*, and *Holdemanella*. The metagenomic sequencing showed that the *Subdoligranulum_sp._APC924/74*, *Streptococcus_parasanguinis*, and *Streptococcus_salivarius* were significantly increased in abundance in the OA group compared to those in the KBD group, and the species *Prevotella_copri*, *Prevotella_sp._CAG:386*, and *Prevotella_stercorea* were significantly decreased in abundance in the OA group compared to those in the KBD group by using metagenomic sequencing.

**Conclusion:**

Our study provides a comprehensive landscape of the gut microbiota between OA and KBD patients and provides clues for better understanding the mechanisms underlying the pathogenesis of OA and KBD.

**Supplementary Information:**

The online version contains supplementary material available at 10.1186/s13075-022-02819-5.

## Background

Osteoarthritis (OA) is a chronic degenerative osteoarthropathy. OA is the most prevalent joint disease leading to the most common disability, which is characterized by cartilage degradation, and abnormal bone formation [1]. Kashin-Beck disease (KBD) is an endemic osteoarthropathy in which affected individuals have shortened and enlarged fingers, deformed limb joints, limited movement, and even dwarfism in some advanced cases [2, 3]. The clinical manifestations of these two diseases are similar and include serious joint pain, limited movement, and deformities of different joints, such as the hands, knees, and ankles [4]. Unlike OA, which mainly causes damage to the articular cartilage of elderly people, the onset age of KBD is 3–12 years old [2]. In addition, KBD and OA are also different in their X-ray images and pathological features [5]. With unknown pathogenesis and limited effective treatments, KBD and OA frequently develop to substantial disability. Therefore, a comparison of the pathogenetic similarities and differences between KBD and OA could help distinguish biological mechanisms and cartilage degeneration processes between KBD and OA.

The gut microbiota is the complex and dynamic microbial population existing in the human intestine. The gut microbiota maintains the balance of the human intestinal microbial ecosystem through its 10^14^ resident microorganisms and more than 1000 bacterial species [6]. In recent years, the composition of the gut microbiota and the concept of a gut-microbiota-cartilage axis participating in the occurrence and development of osteochondral diseases have attracted extensive attention. A number of studies have demonstrated the linkage between the dysbiosis of the gut microbiota and OA in animal models and humans [7–9]. Cartilage injuries of KBD are caused by the interaction between genetic and environmental factors, mostly *Fusarium* mycotoxins and selenium deficiency, which could be linked through the gut microbiome [10, 11]. However, a comparison of gut microbiota composition between OA and KBD has never been reported.

Hence, we investigated the differences in the microbiome between KBD and OA by 16S rDNA gene sequencing and metagenomic sequencing. The results provide a comprehensive landscape of the gut microbiota in KBD and OA patients, which could partly elucidate the biological mechanism and cartilage degeneration processes of KBD and OA.

## Methods

### Study design and sample collection

This study recruited 32 KBD patients from Xunyi County, one of the severe KBD endemic areas in China. KBD patients were diagnosed strictly according to the national diagnostic criteria of KBD in China [WS/T 207-2010]. Correspondingly, 32 OA patients were recruited from Xi’an Hong Hui Hospital and diagnosed strictly according to the Modified Outerbridge Classification. Subjects were diagnosed with KBD when manifested with X-ray alterations, such as defects and sclerosis on the bone end of phalanges combined with compression changes of the calcaneus and talus, and enlarged/deformed limb joints (hand, elbow, knee, ankle, etc.). The subjects were excluded for the following situations: they were suffering or had previously suffered from any other osteoarticular diseases (such as rheumatoid arthritis, gout, skeletal fluorosis) or any other type of macrosomia, disorder of osteochondrodysplasia, or chronic diseases (such as hypertension, diabetes, coronary heart diseases, etc.), or accepted any treatment in recent 6 months, or had a history of inflammatory bowel disease (IBD), or irritable bowel syndrome (IBS); and patients with complications of complete intestinal obstruction. In addition, patients using antibiotics, probiotics, prebiotics, or synbiotics within 2 months of sampling were also excluded. All recruited patients with KBD and OA were local residents in Shaanxi province’s Guanzhong region, where the people mainly eat wheat products and green vegetables three meals a day. In addition, before inclusion, we conducted a 5-day semi-quantitative dietary questionnaire in face-to-face interviews. Patients were excluded if they changed eating habits in recent 5 days, such as drank unusual amount of alcohol and consumed vast quantities of meat and spicy or pickled food. This study has been approved by the human ethics committee of Xi’an Jiaotong University (NO. 2018-206). All subjects signed a written informed consent form. Their general clinical data were recorded, including age, gender, educational background, body mass index (BMI), and disease degree. All patients were Shaanxi Han Chinese with similar geographic areas and eating habits. All qualified stool samples were self-sampled prior to mechanical feces preparation and were transported immediately to the laboratory, divided into three portions per sample, packed into three freezer tubes, frozen in liquid nitrogen overnight, and preserved at −80°C for further testing.

### DNA extractions and 16S rDNA gene sequencing

DNA from different samples was extracted using The E.Z.N.A.® Stool DNA Kit (D4015-02, Omega, Inc., USA) according to the manufacturer’s instructions. The reagent which was designed to uncover DNA from trace amounts of the sample has been shown to be effective for the preparation of DNA of most bacteria. Sample blanks consisted of unused swabs processed through DNA extraction and tested to contain no DNA amplicons. The total DNA was eluted in 50 μL of Elution buffer by a modification of the procedure described by the manufacturer (QIAGEN) and stored at −80°C until measurement in the PCR by LC-BIO TECHNOLOGIES (HANGZHOU) CO., LTD., Hang Zhou, Zhejiang Province, China.

The V3-V4 region of the prokaryotic (bacterial and archaeal) small-subunit (16S) rRNA gene was amplified with primers 341F (5′-CCTACGGGNGGCWGCAG-3′) and 805R (5′-GACTACHVGGGTATCTAATCC-3′). The 5′ ends of the primers were tagged with specific barcodes per sample and sequencing universal primers. PCR amplification was performed in a total volume of 25-μL reaction mixture containing 25 ng of template DNA, 12.5 μL PCR Premix, 2.5 μL of each primer, and PCR-grade water to adjust the volume. The PCR conditions to amplify the prokaryotic 16S fragments consisted of an initial denaturation at 98°C for 30 s; 32cycles of denaturation at 98°C for 10 s, annealing at 54°C for 30 s, and extension at 72°C for 45 s; and then final extension at 72°C for 10 min. The PCR products were confirmed with 2% agarose gel electrophoresis. Throughout the DNA extraction process, ultrapure water, instead of a sample solution, was used to exclude the possibility of false-positive PCR results as a negative control. The PCR products were purified by AMPure XT beads (Beckman Coulter Genomics, Danvers, MA, USA) and quantified by Qubit (Invitrogen, USA). The amplicon pools were prepared for sequencing and the size and quantity of the amplicon library were assessed on Agilent 2100 Bioanalyzer (Agilent, USA) and with the Library Quantification Kit for Illumina (Kapa Biosciences, Woburn, MA, USA), respectively. The libraries were sequenced on the NovaSeq PE250 platform.

Samples were sequenced on an Illumina NovaSeq platform according to the manufacturer’s recommendations, provided by LC-Bio. Paired-end reads were assigned to samples based on their unique barcode and truncated by cutting off the barcode and primer sequence. Paired-end reads were merged using FLASH. Quality filtering on the raw reads was performed under specific filtering conditions to obtain the high-quality clean tags according to the fqtrim (v0.94). Chimeric sequences were filtered using Vsearch software (v2.3.4). After dereplication using DADA2, we obtained the feature table and feature sequence. Alpha diversity and beta diversity were calculated by QIIME2, which the same number of sequences was extracted randomly through reducing the number of sequences to the minimum of some samples, and the relative abundance (X bacteria count/total count) is used in bacteria taxonomy. Alpha diversity and beta diversity were analyzed by the QIIME2 process, and pictures were drawn by R (v3.5.2). The sequence alignment of species annotation was performed by Blast, and the alignment database was SILVA and NT-16S.

### Metagenomic sequencing

The DNA of the microbiota in fecal samples from 32 KBD and 32 OA were used to perform metagenomics analysis. DNA library was constructed by TruSeq Nano DNA LT Library Preparation Kit (FC-121-4001). DNA was fragmented by dsDNA Fragmentase (NEB, M0348S) by incubation at 37°C for 30min. Library construction begins with fragmented cDNA. Blunt-end DNA fragments are generated using a combination of fill-in reactions and exonuclease activity, and size selection is performed with provided sample purification beads. An A-base is then added to the blunt ends of each strand, preparing them for ligation to the indexed adapters. Each adapter contains a T-base overhang for ligating the adapter to the A-tailed fragmented DNA. These adapters contain the full complement of sequencing primer hybridization sites for single, paired-end, and indexed reads. Single- or dual-index adapters are ligated to the fragments, and the ligated products are amplified with PCR by the following conditions: initial denaturation at 95°C for 3 min; 8 cycles of denaturation at 98°C for 15 s, annealing at 60°C for 15 s, and extension at 72°C for 30 s; and then final extension at 72°C for 5 min.

Raw sequencing reads were processed to obtain valid reads for further analysis. First, sequencing adapters were removed from sequencing reads using cutadapt v1.9. Secondly, low-quality reads were trimmed by fqtrim v0.94 using a sliding-window algorithm. Thirdly, reads were aligned to the host genome using bowtie2 v2.2.0 to remove host contamination. Once quality-filtered reads were obtained, they were de novo assembled to construct the metagenome for each sample by IDBA-UD v1.1.1. All coding regions (CDS) of metagenomic contigs were predicted by MetaGeneMark v3.26. CDS sequences of all samples were clustered by CD-HIT v4.6.1 to obtain unigenes. Unigene abundance for a certain sample was estimated by TPM based on the number of aligned reads by bowtie2 v2.2.0. The lowest common ancestor taxonomy of unigenes was obtained by aligning them against the NCBI NR database by DIAMOND v 0.9.14. Similarly, the functional annotation of unigenes was obtained. Based on the taxonomic and functional annotation of unigenes, along with the abundance profile of unigenes, the differential analysis was carried out at each taxonomic or functional or gene-wise level by Fisher’s exact test.

### Statistical analyses

Significant differences in clinical characteristics were evaluated with Pearson’s chi-square test or Fisher’s exact test. Spearman’s correlation analysis was conducted to calculate the correlation between species and metabolites. Differences were considered significant when *p* < 0.05. All data were analyzed with GraphPad Prism 6 software (GraphPad software, Inc., San Diego, CA, USA), R version 3.5.2 (R Foundation for Statistical Computing, Vienna, Austria), and Microsoft Excel (Microsoft Corporation, Seattle, WA, USA).

## Results

### Clinical characteristics of the population

All subjects were Han Chinese from Shaanxi Province in this study. They had similar eating habits to control dietary differences. The demographic parameters were generally similar between the two groups. The average age of KBD patients was 62 years and that of OA patients was 67 years. There was no significant difference in age, sex ratio, or BMI between the two groups (Table S1).

### Alterations in gut microbiota composition in patients with OA and KBD based on 16S rDNA analysis

Our present microbiome study obtained 3,721,237 high-quality 16S rDNA reads with a median of 58,566 reads (range from 37,346 to 91,295) per sample (Supplementary Table S2). This sequencing generated 9316 features from 64 samples (Supplementary Table S3). Detailed information about the 16S rDNA data from all samples is provided in Supplementary Table S4.

### Comparative analysis of gut microbiota composition in patients with OA and KBD

**Fig. 1:**
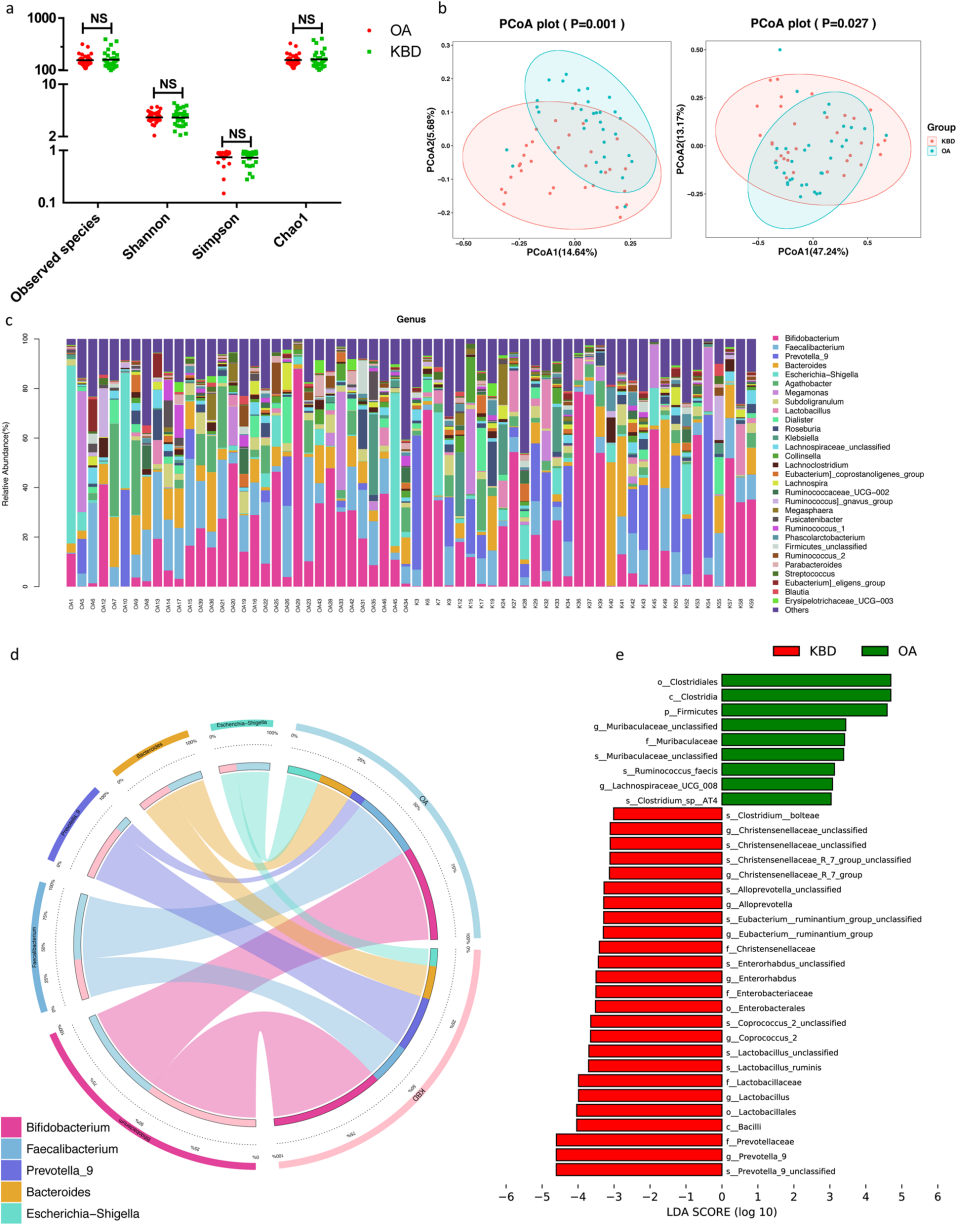
Gut microbiome diversity and structure analysis based on 16S rDNA sequencing data. **a** Species diversity differences between the OA and KBD groups were estimated by the observed species, Shannon, Simpson, and Chao1 indices. NS, not significant; OA, patient in the osteoarthritis group; KBD, patient in the KBD group. **b** Principal coordinates analysis (PCoA) of the microbiota based on the unweighted (*P* = 0.001) and weighted (*P* = 0.027) UniFrac distance matrices for the OA and KBD groups. **c** Component proportions of the bacterial genus in each group; *n* = 32 for the OA group and *n* = 32 for the KBD group. **d** Circos plot. The left side of the circle represents species and the right side represents sample groups; different colors represent different taxonomic categories and sample groups. From left to right, the thickness of the same color line in the inner ring represents the relative abundance of the species in different sample groups; from right to left, the thickness of the same color line in the inner ring represents the proportion of different species in the sample group. **e** Linear discriminant analysis (LDA) integrated with effect size (LEfSe). The differences in abundance between the OA and KBD groups

Alpha diversity and beta diversity were compared between OA and KBD subjects to evaluate the characterization of the OA-associated gut microbiome. There were no significant differences in the Shannon, observed species, and Chao1 indices (Fig. 1a and Supplementary Table S5). The Venn diagram displayed 3049 unique features in the OA group and 2987 in the KBD group, whereas both groups shared 1216 features (Supplementary Fig. S1a). Principal coordinates analysis (PCoA) was performed to investigate the extent of the similarity of the microbial communities between the two groups based on unweighted and weighted UniFrac distance metrics (Fig. 1b). The analysis indicated that the microbiota composition of OA group clusters was more heterogeneous and significantly different from that of the KBD group.

The relative proportions of dominant taxa at the genus level were assessed by microbial taxon assignment between the OA and KBD groups. We observed considerable variability in the gut microbiota across samples in each group (Fig. 1c). The assessment identified 415 genera in each group. *Bifidobacterium* was the most predominant genus, accounting for 20.14% and 23.98% of the features in the OA and KBD groups, respectively. In addition, *Faecalibacterium* (14.01% versus 8.58%) and *Escherichia-Shigella* (7.08% versus 3.69%) were enriched in the OA group compared to the KBD group, and *Prevotella_9* (11.70% versus 3.01%) was enriched in the KBD group compared to the OA group (Fig. 1d and Supplementary Table S6).

To compare the differences in the fecal microflora between the two groups, the Wilcox test was performed for different classification levels. At the phylum level, the OA group was characterized by higher *Epsilonbacteraeota* and *Firmicutes* levels (Supplementary Fig. S1b). At the genus level, 52 genera were identified differentially abundant between the two groups (Table S7). Of these discriminatory taxa, *Raoultella* (*p* = 0.047), *Citrobacter* (*p* = 0.0029), *Flavonifractor* (*p* = 0.0034), *g__Lachnospiraceae_UCG-004* (*p* = 0.0295), and *Burkholderia-Caballeronia-Paraburkholderia* (*p* = 0.0001) were found to be more abundant in the OA group than in the KBD group. Meanwhile, *Prevotella_9* (*p* = 0.002), *Lactobacillus* (*p* = 0.0018), *Coprococcus_2* (*p* = 0.0084), *Senegalimassilia* (*p* = 0.004), and *Holdemanella* (*p* = 0.0065) were found to be more abundant in the KBD group than in the OA group (Supplementary Fig. S1c).

Linear discriminant analysis (LDA) effect size (LEfSe) was performed to generate a cladogram to identify the specific bacteria associated with OA (Supplementary Fig. S1d). LDA distribution diagram analysis (LDA score > 3) showed a clear alteration of the microbiota characterized by higher *Clostridiales* and *Firmicutes* levels in the OA group (Fig. 1e). The genera *Muribaculaceae_unclassified* and *Lachnospiraceae_UCG_008* were more abundant in the OA group, while the genera *Prevotella_9*, *Lactobacillus*, *Coprococcus_2*, *Alloprevotella, Enterorhabdus*, and *Christensenellaceae_R_7_group* were more abundant in the KBD group (Fig. 1e).

A random forest model based on the differentially abundant genera was used to test whether potential diagnostic biomarkers could be used to predict the OA and KBD groups. The optimal model utilized 20 genera that provided the best discriminatory power (Supplementary Fig. 1e). Based on the above analysis, the distribution of the microbial community showed significant differences between OA and KBD subjects. Then, to explore the potential value of the identified bacterial biomarkers for the discrimination of OA and KBD, we produced receiver operating characteristic (ROC) curves and computed the area under the curve (AUC) values. The top 3 AUC values were 87.10% for *Delftia*, 83.59% for *Burkholderia_Caballeronia_Paraburkhoderia*, and 79.00% for *Muribaculaceae_unclassified* (Supplementary Fig. 1f). These results confirmed that gut-microbiota-based biomarkers could distinguish OA and KBD patients with high possibility and reliability for differential diagnosis.

### Metagenomic sequencing further revealed significant differences between the OA and KBD

**Fig. 2:**
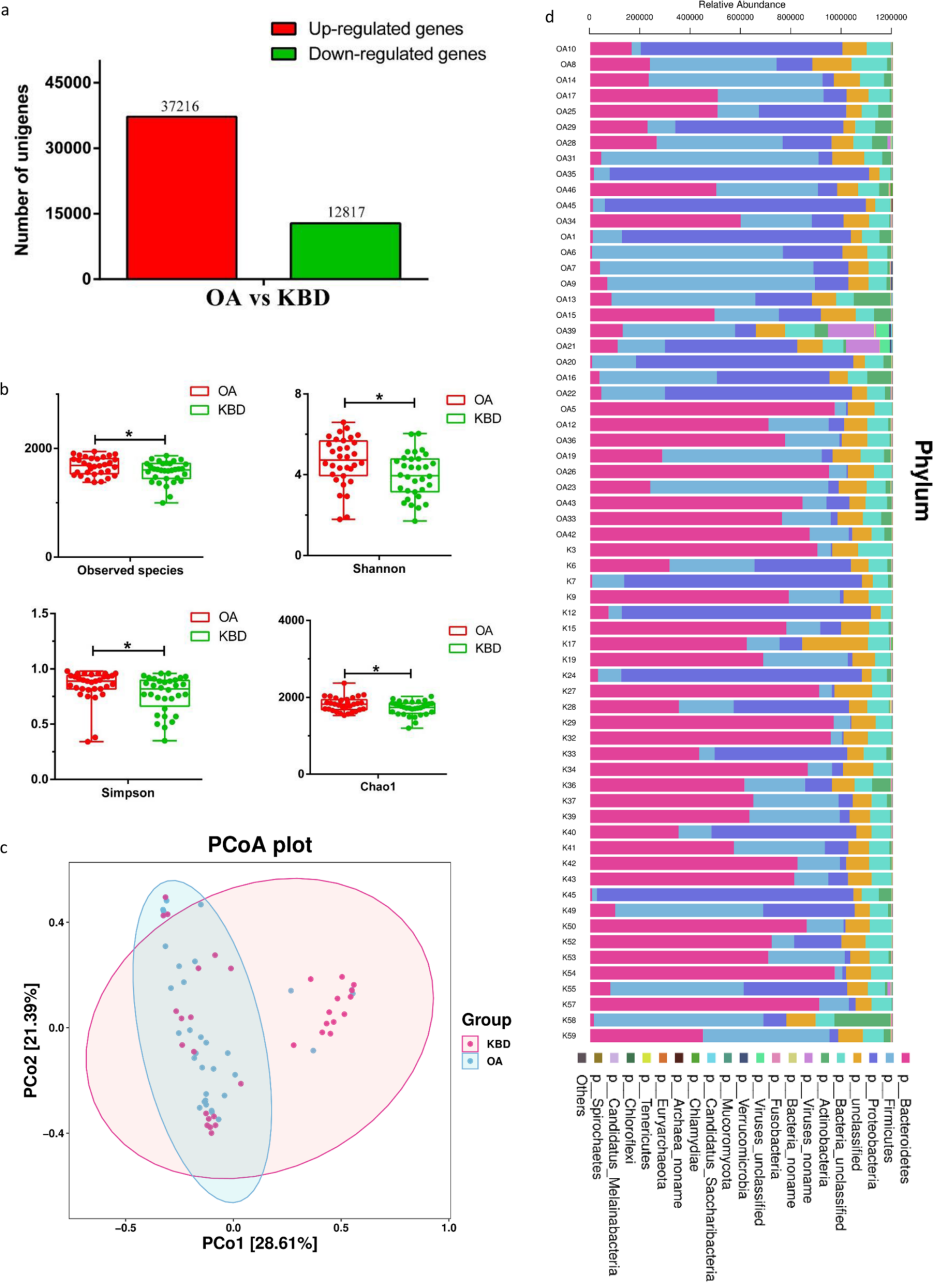
The gut microbiota differences in OA and KBD groups based on the metagenomic sequencing data. **a** Differentially expressed unigenes between OA and KBD groups. **b** Alpha diversity differences between the OA and KBD groups were estimated by the observed species, Shannon, Simpson, and Chao1 indices. *P<0.05. OA, patients with OA group; KBD, patients with KBD group. **c** The PCoA analysis based on the Bray-Curtis distance matrix between the OA and KBD groups at the species level (*P* = 0.001). **d** Component proportions of phylum in each group; *n* = 32 for the OA group and *n* = 32 for the KBD group based on the metagenomic sequencing data

We performed metagenomic sequencing on fecal samples to compare the differences in cartilage injuries and gut microbiota between OA and KBD and to identify whether gut microbial changes at the species level are associated with the responsive genes or functions of the gut bacteria in OA and KBD patients. We predicted 154,367 genes (Supplementary Fig. S2 a–d). OA samples contained 5498 specific genes compared to KBD samples (Supplementary Fig. S2 e). In addition, 50,033 differentially expressed unigenes (37,216 upregulated and 12,817 downregulated) were identified in the OA group compared to the KBD group (Fig. 2a).

By measuring the Shannon, observed species, Simpson, and Chao1 indices, it was found that the alpha diversity was significantly higher in the OA group than in the KBD group (Fig. 2b). The PCoA based on the Bray-Curtis distance matrix revealed striking differences in microbial composition between the OA and KBD groups at the species level (Fig. 2c).

**Fig. 3:**
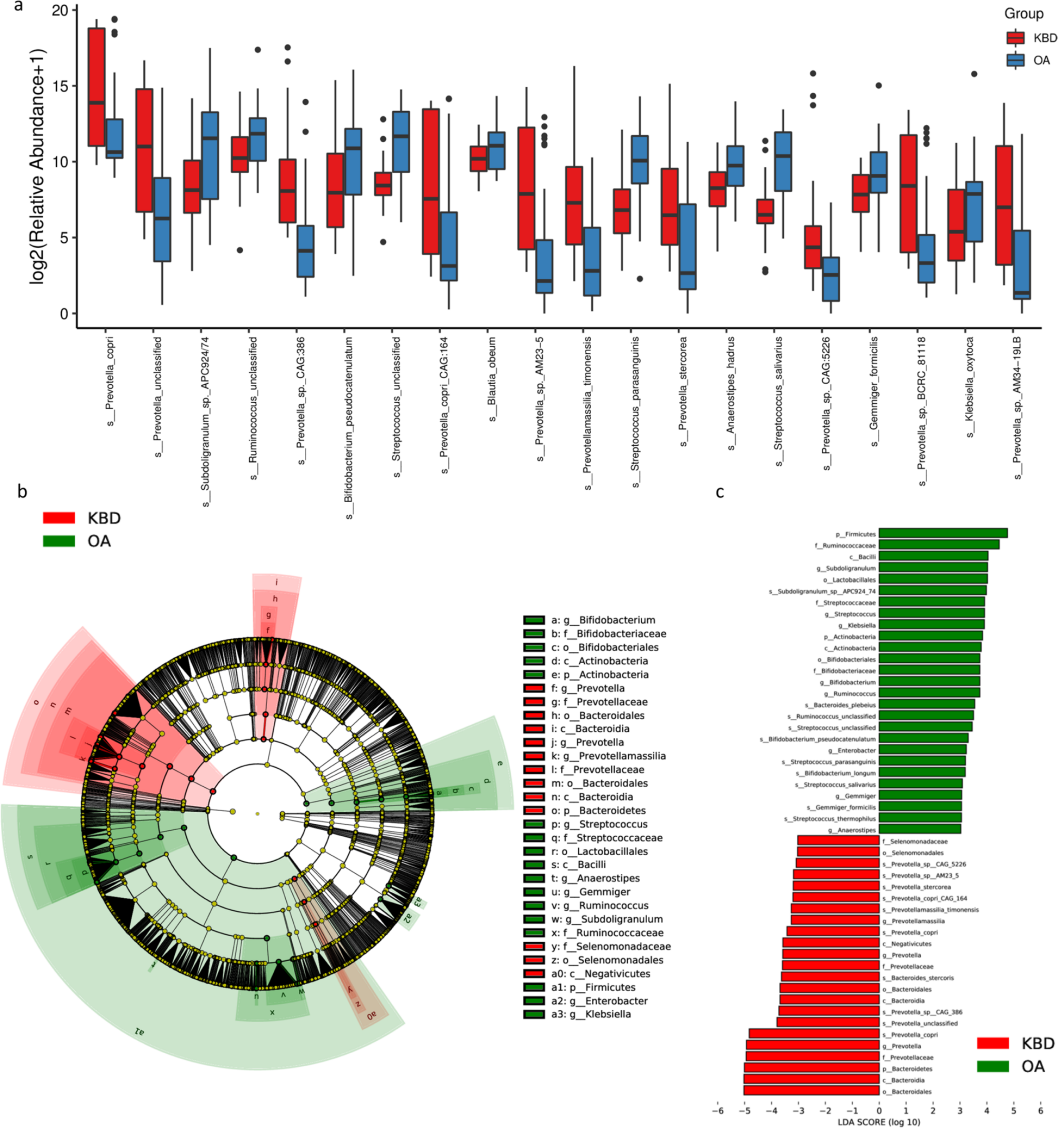
The gut microbiota differences in OA and KBD groups based on the metagenomic sequencing data. **a** The relative abundance of top 20 species enriched in OA versus KBD. The box represents the interquartile ranges, inner line denotes the median. **b** Cladogram indicating the phylogenetic distribution of microbiota correlated with the OA and KBD groups. **c** Linear discriminant analysis (LDA) integrated with effect size (LEfSe). The differences in abundance between the OA and KBD groups

The relative proportions of dominant taxa at the phylum level were assessed by microbial taxon assignment in both groups. We observed considerable variability in the gut microbiota across samples in each group (Fig. 2d), and the distributions of the abundances of *Bacteroidetes*, *Firmicutes*, and *Proteobacteria* were different between KBD and OA. Then, we compared the profile differences and identified 695 species with differential relative abundance between the OA and KBD groups (Supplementary Table S8). The abundances of the species *Prevotella_copri*, *Prevotella_sp._CAG:386*, and *Prevotella_stercorea* were significantly decreased in the OA group compared to the KBD group (Fig. 3a, Table S8). *Subdoligranulum_sp._APC924/74*, *Streptococcus_parasanguinis*, and *Streptococcus_salivarius* were significantly increased in abundance in the OA group compared to the KBD group (Fig. 3a, Table S8). Consistent with the 16S rRNA analysis at the genera level, most of the identified differentially abundant species belonged to the genera *Prevotella* and *Streptococcus*.

Linear discriminant analysis (LDA) effect size (LEfSe) analysis was performed to generate a cladogram to identify the specific bacteria associated with OA and KBD (Fig. 3b), and LDA distribution diagram analysis (LDA score > 3) showed the same results as the cladogram. A clear alteration of the characterized microbiota was higher *Bacteroidetes* and *Prevotella 9* levels in the KBD group (Fig. 3c). However, *Firmicutes* levels were significantly increased in the OA group (Fig. 3c). The genera *Subdoligranulum, Streptococcus*, *Bifidobacterium*, and *Ruminococcus* were more abundant in the OA group, while the genera *Prevotellamassilia_timonensis*, *Prevotellamassilia*, and *Prevotella* were more abundant in the KBD group (Fig. 3c).

### Functional analysis of metagenomic sequencing revealed disrupted bacterial functions between the OA and KBD groups

**Fig. 4:**
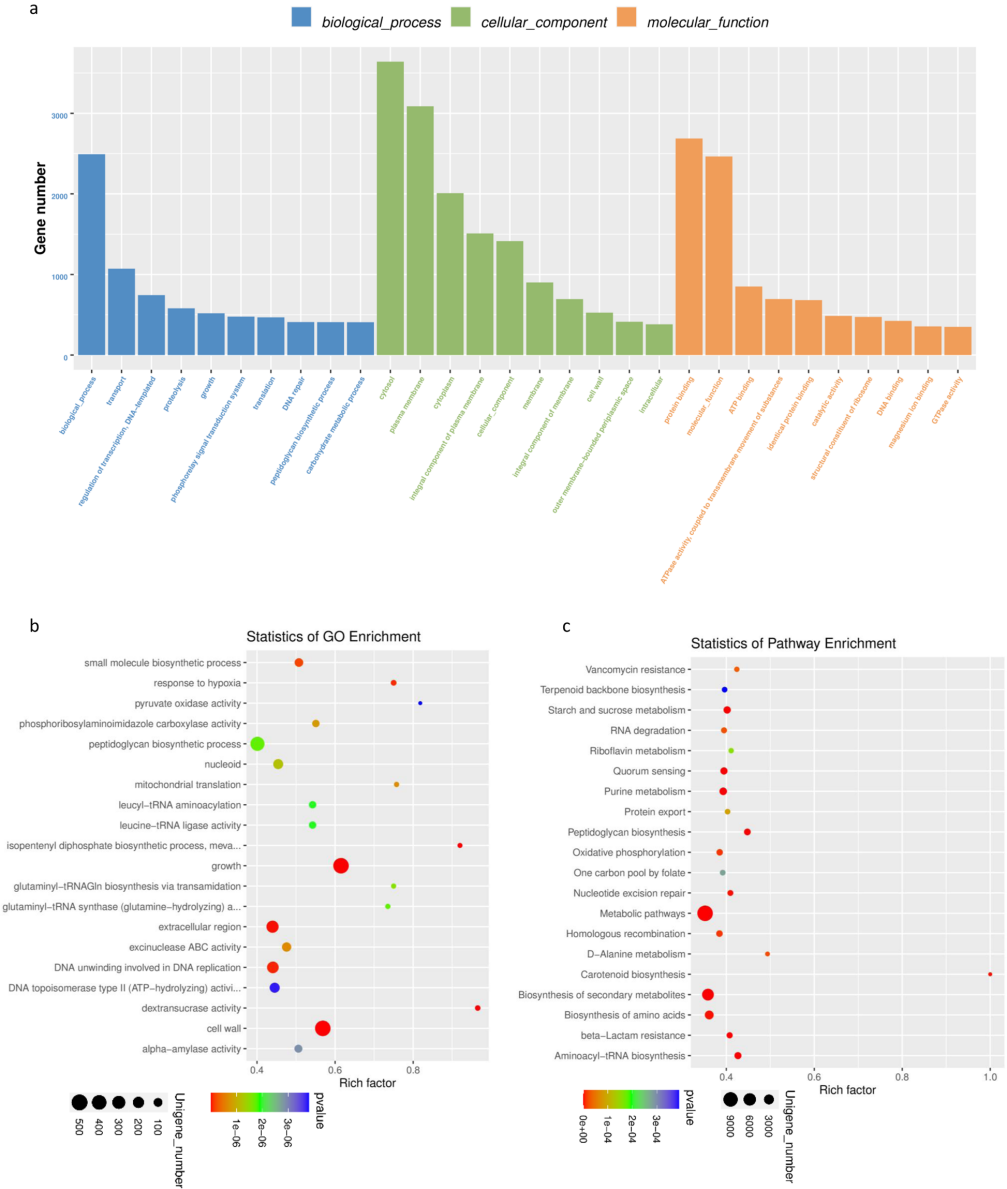
**a** GO function classification analysis of differentially expressed unigenes between OA and KBD group. **b** GO enrichment analysis of differentially expressed unigenes between OA and KBD groups. **c** Pathway classification based on KEGG enrichment analysis of differentially expressed unigenes between OA and KBD groups

To describe the function of differentially expressed unigenes from different groups, we selected the top 10 GO items of the three forms (biological process, cellular component, and molecular function) of definitions by the GO database. The results of the GO function classification analysis of differentially expressed unigenes between the OA and KBD groups are shown in Fig. 4a. GO enrichment analysis of differentially expressed unigenes in different groups was performed, and the top 20 GO terms are shown in Fig. 4b. KEGG analysis of differentially expressed unigenes showed the most abundant pathways in different groups. Peptidoglycan biosynthesis, aminoacyl-tRNA biosynthesis, metabolic pathways, and starch and sucrose metabolism differed between the OA and KBD groups (Fig. 4c and Supplementary Table S9). The analysis further suggested that these disrupted pathways in different groups might contribute to the potential differences between OA and KBD patients that were generated by the dysbiosis of the gut microbiome and metabolites.

## Discussion

The concept of a gut-microbiome-cartilage axis has been proposed for a few years. Several studies [7–9] have identified the linkage between the gut microbiota and OA by establishing correlations between joint injury and serum levels of bacterial metabolites. We previously found a comprehensive landscape of the gut microbiota in KBD patients [12]. However, at present, no systematic study has clearly described the alterations between OA and KBD in the gut microbiota. In this study, we provided the first evidence of the microbiome of fecal samples from patients with OA and KBD, which revealed microbial diversity and the association between OA and KBD and the gut microbiota.

Recently, members of the gut microbiome were found in human cartilage. OA cartilage samples were dominated by *Betaproteobacteria*, while control samples were characterized by *Actinobacteria* and *Clostridia* [13]. *Bacteroides* has been detected in 183 samples of either RA or OA synovium and synovial fluid [14]. Therefore, the translocation of the gut microbiota might occur in the subchondral bone marrow and/or deeper zone of cartilage, which could directly cause OA changes, as has been proposed by researchers [15]. However, it is still a conundrum how these bacteria can reach the cartilage, meaning the subchondral bone marrow, deep cartilage vessels, cartilage matrix, and chondrocytes. This translocation might disturb cartilage metabolism through gut microbiota metabolic products, and some metabolites might be toxic to cartilage [15]. Primary cartilage loss is the main pathological change difference between OA and KBD. OA is characterized primarily by progressive degradation starting in the superficial zone, with subsequent inflammation. Angiogenesis occurs at the early stage of OA in the osteochondral plate. This process could improve the translocation of living bacteria and their products to the deeper zone of cartilage. Low-grade inflammation and cartilage degradation might be induced by the microbial antigens in the deeper layers of cartilage, as pathogenic bacterial antigens, which can produce the production of proinflammatory mediators in chondrocytes [16]. Unlike OA, characteristic necrosis of chondrocytes in KBD starts primarily in the deep zone of articular cartilage and the hypertrophic layer of epiphyseal plate cartilage. Therefore, such transient translocation of the gut microbiota to the subchondral bone marrow or deeper zones of cartilage might also contribute to explaining KBD cartilage loss.

Recently, it has been proven that microbiota translocation could occur in the deep zone of cartilage, and the complex chemical substances presented by dietary and host nutrients can be converted into metabolites [15], which could affect cartilage metabolism by its toxicity and injury functions [17]. In our previous study, many fatty acids, such as gamma-linolenic acid, palmitelaidic acid, 3-oxoctadecanoic acid, and oleic acid, were identified in the serum from patients with KBD. Docosapentaenoic acid, palmitelaidic acid, gamma-linolenic acid, oleic acid, and (E)-13-hydroxy-10-oxo-11-octadecenoic acid were negatively correlated with the abundance of Paenibacillus montanisoli and positively correlated with the abundance of Buttiauxella brennerae in KBD. LysoPE 20:5, LysoPC 20:5, and LysoPI 20:5 were negatively correlated with the abundance of the species Parvimonas sp. KA00067 and positively correlated with the abundance of the species Rothia sp. HMSC065C03 [12]. The evidence above suggests that gut microbiota dysbiosis is closely correlated with KBD development and that lipid metabolism dysregulation might be a crucial factor.

Short-chain fatty acids (SCFAs) are mainly produced by gut bacteria, and SCFAs could have a crucial role in maintaining bone homeostasis, downregulating the proinflammatory stimuli sustained by regulatory T cells, and inhibiting bone resorption through direct inhibition of osteoclast activity [18]. Boer et al. found that the abundance of *Streptococcus* species was significantly associated with increased knee pain and effusion severity in the joint. They proposed that greater *Streptococcus* abundance might cause an increase in bacterial products in the circulation through increased production of metabolites that pass the gut-blood barrier [19]. *Streptococcus* has been reported previously in osteomyelitis, rheumatic fever, and poststreptococcal reactive arthritis [20–25]. Several *Streptococcus* spp. produced membrane vesicles [26], which might release immunogenic products [27, 28] that can drive macrophage activation by TLR pathways. This macrophage activation process is considered to be involved in OA-related pain and joint inflammation [28–30]. *Streptococcus* are major lactate producers in the gut [31], and it was shown that high concentrations of lactate induced chondrocyte apoptosis and that it accumulated in chondrocyte from patients with OA [32]. In our study, *Streptococcus* was more predominant in OA patients than in KBD patients. At the species level, *Streptococcus_parasanguinis* and *Streptococcus_salivarius* were significantly increased in abundance in the OA group compared to the KBD group in our study.

*Prevotella* is one of the most dominant bacteria in the human intestinal tract [33, 34]. *Prevotella* has been reported to be capable of producing succinic acid, which participates in polysaccharide synthesis [35] and could improve the immune response to protect host health [36]. Immune dysfunction has been considered to be involved in the pathogenesis of KBD [37–39]. In this study, the abundances of the species *Prevotella_copri*, *Prevotella_sp._CAG:386*, and *Prevotella_stercorea* were significantly increased in the KBD group compared to the OA group. In another study, *Prevotella* was found to be more predominant in the RA group than in the OA group [33]. In addition, *Prevotella_copri* was significantly expanded in RA patients compared with a healthy group [14]. *Prevotella_9* are major acetate producers in the gut [31], and it has been proven that acetic acid and its compounds (iodoacetic acid, etc.) can lead to chondrocyte injury by changing the expression of type II collagen, matrix metalloproteinase, and related inflammatory response factors in chondrocytes [40]. Therefore, a significant decrease in *Prevotella* might provide some clues underlying the role of gut microbiota in the mechanisms of OA, while a significant increase in *Prevotella* may reveal new pathogenic evidence for KBD.

Gut microbiota dysbiosis has been previously proven to be correlated with aging. It showed a decrease in anti-inflammatory species but an increasing trend of proinflammatory species [41]. The correlation between microbiota and OA could be considered the link between cartilage degeneration and metabolites in serum produced by gut microbiota [9]. Some mouse OA models and human OA studies verified the above correlation by increasing the level of circulatory inflammatory markers, such as lipopolysaccharides [42, 43]. Therefore, the proinflammatory metabolites induced by the gut microbiota might play a key role in OA pathogenesis. Unlike OA, cartilage damage in patients with KBD is most likely driven by the interaction between genetic and environmental factors, such as *Fusarium* mycotoxins and selenium. The selenium deficiency status in KBD patients caused by environmental low selenium could affect the composition of intestinal flora. In turn, translocated *Fusarium* mycotoxins and harmful bacteria from the intestinal tract to the subchondral bone marrow enter the deep zone of articular cartilage and hypertrophic layers of epiphyseal plate cartilage, which are the primary lesion sites of KBD. Finally, these risk factors trigger the responsive genes of chondrocytes and then directly contribute to KBD cartilage damage, such as apoptosis and necrosis.

## Conclusions

In conclusion, we comparatively revealed the different compositions of the gut microbiota in OA and KBD patients. The altered genera, phyla, and species in OA and KBD were identified, and the findings could provide clues for a better understanding of the mechanisms underlying the pathogenesis of OA and KBD and how the gut microbiota might participate in the original cartilage loss OA and KBD.

## Supplementary Information

**Additional file 1: Figure S1**. Comparative gut microbiome diversity and structure analysis between OA and KBD. (a) Venn diagram of the observed features in OA and KBD. (b) Wilcox-test results for evaluating the relative abundance of significantly different microbiota at the phylum level. (c) Wilcox-test results for evaluating the relative abundance of significantly different microbiota at the genus level. (d) Cladogram indicating the phylogenetic distribution of microbiota correlated with the OA and KBD group. (e) Classification performance of a random forest model using 16s rRNA genus abundance assessed by R random Forest package. (f) ROC curve displaying the top 3 biomarkers for classification between OA and KBD. AUC, area under curve.

**Additional file 2: Figure S2**. The information of gene catalogue based on the metagenomic sequencing. (a) The distribution of UniqGene length, (b) The dilution curve of core genes, (c) The dilution curve of pan genes. (d-f) Venn diagrams demonstrate the number of altered genes shared between OA and KBD group.

**Additional file 3: Table S1**. Characteristics of participants in this study.

**Additional file 4: Table S2**.

**Additional file 5: Table S3**.

**Additional file 6: Table S4**.

**Additional file 7: Table S5**.

**Additional file 8: Table S6**.

**Additional file 9: Table S7**.

**Additional file 10: Table S8**.

**Additional file 11: Table S9**.

## Data Availability

The dataset supporting the conclusions of this article is included within the article and its additional files.

## Abbreviations

KBD: Kashin-Beck disease
OA: Osteoarthritis
RA: Rheumatoid arthritis
GO: Gene ontology
IBS: Irritable bowel syndrome
IBD: Inflammatory bowel disease
CRC: Colorectal cancer
PCoA: Principal coordinates analysis
LDA: Linear discriminant analysis
LEfSe: Effect size
ROC: Receiver operating characteristic
AUC: Area under the curve
KEGG: Kyoto Encyclopedia of Genes and Genomes
