# Supplementary figures and images for: Comparative analysis of the gut microbiota composition between knee osteoarthritis and Kashin-Beck disease in Northwest China
Source: Arthritis Res Ther. 2022 May 30;24:129. doi: 10.1186/s13075-022-02819-5 (PMC9150333; doi:10.1186/s13075-022-02819-5)

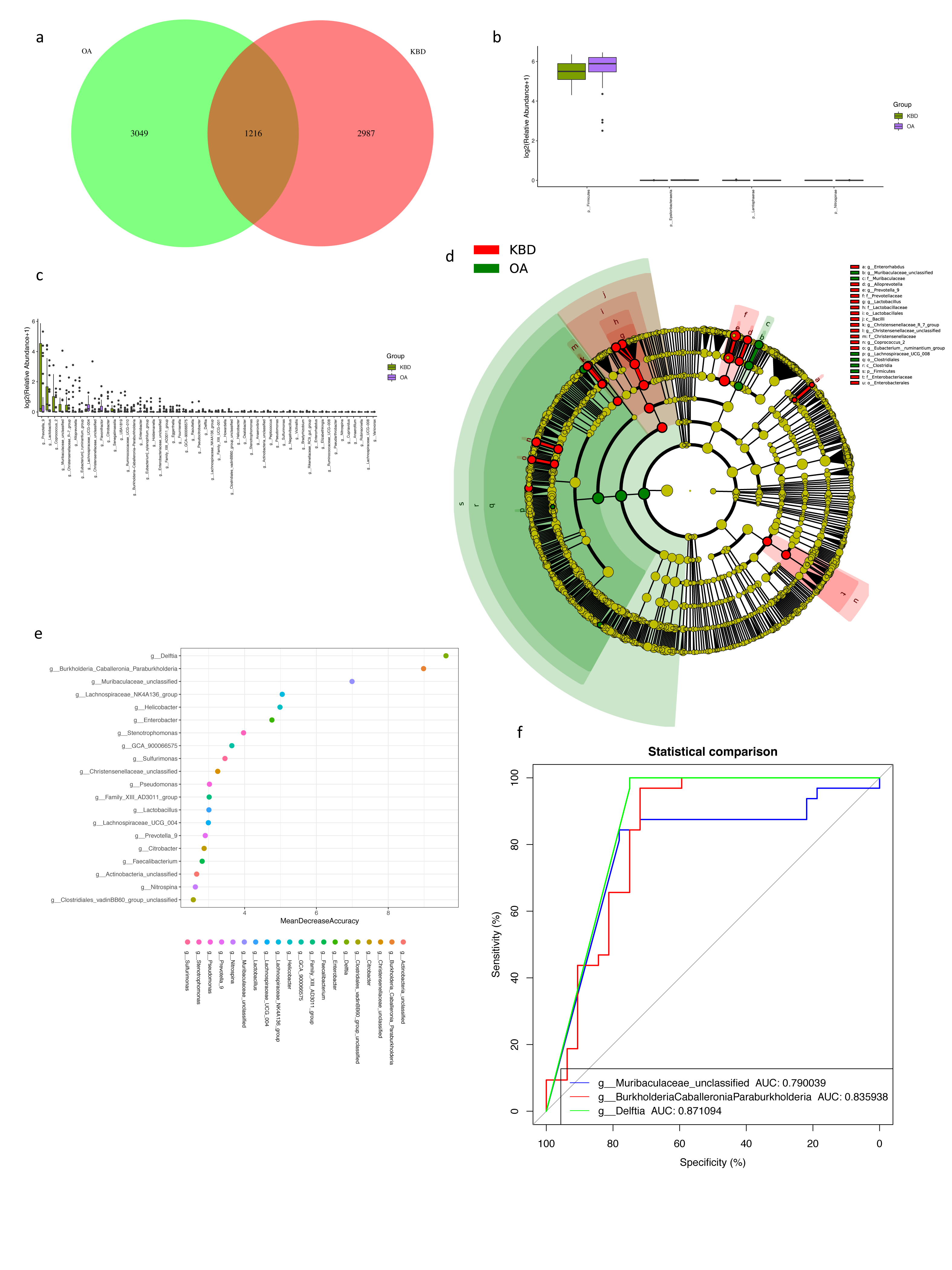

Supplement: Supplementary file 1 — Additional file 1: Figure S1. Comparative gut microbiome diversity and structure analysis between OA and KBD. (a) Venn diagram of the observed features in OA and KBD. (b) Wilcox-test results for evaluating the relative abundance of significantly different microbiota at the phylum level. (c) Wilcox-test results for evaluating the relative abundance of significantly different microbiota at the genus level. (d) Cladogram indicating the phylogenetic distribution of microbiota correlated with the OA and KBD group. (e) Classification performance of a random forest model using 16s rRNA genus abundance assessed by R random Forest package. (f) ROC curve displaying the top 3 biomarkers for classification between OA and KBD. AUC, area under curve. [file 13075_2022_2819_MOESM1_ESM.tif]

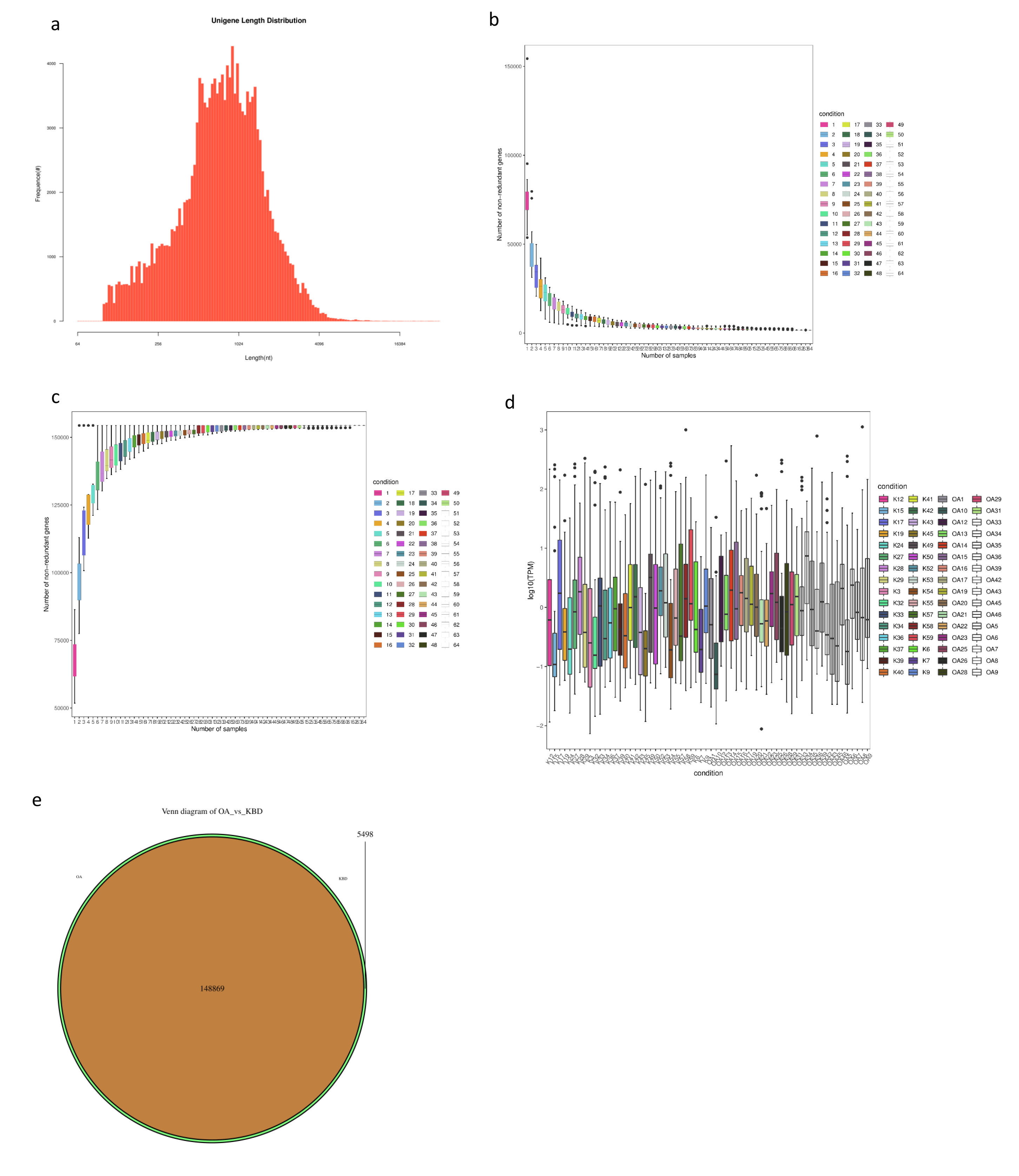

Supplement: Supplementary file 2 — Additional file 2: Figure S2. The information of gene catalogue based on the metagenomic sequencing. (a) The distribution of UniqGene length, (b) The dilution curve of core genes, (c) The dilution curve of pan genes. (d-f) Venn diagrams demonstrate the number of altered genes shared between OA and KBD group. [file 13075_2022_2819_MOESM2_ESM.tif]
