# Supplementary material for: Comparative analysis of the gut microbiota composition between knee osteoarthritis and Kashin-Beck disease in Northwest China
Source: Arthritis Res Ther. 2022 May 30;24:129. doi: 10.1186/s13075-022-02819-5 (PMC9150333; doi:10.1186/s13075-022-02819-5)
Supplement: Supplementary file 3 — Additional file 3: Table S1. Characteristics of participants in this study. [file 13075_2022_2819_MOESM3_ESM.docx]

| Characteristic | KBD | OA | P value |
| --- | --- | --- | --- |
| Subjects (n) | 32 | 32 |  |
| Male/Female | 10/22 | 9/23 | >0.05 |
| Age (mean), years | 62 | 67 | >0.05 |
| Degree | Ⅰ* (16) | Ⅲ* (9) |  |
|  | Ⅱ* (16) | Ⅳ* (23) |  |
| BMI | 23.41 | 24.24 | >0.05 |

Table S1 Characteristics of participants in this study

* grade ⅠandⅡ KBD patient according to the national diagnostic criteria of KBD in China [WS/T 207-2010]; grade Ⅲ and Ⅳ OA patients according to the Kellgren Lawrence scoring system. TKA: total knee arthroplasty
